# Tetramethylpyrazine Produces Antidepressant-Like Effects in Mice Through Promotion of BDNF Signaling Pathway

**DOI:** 10.1093/ijnp/pyv010

**Published:** 2015-03-06

**Authors:** Bo Jiang, Chao Huang, Xiang-Fan Chen, Li-Juan Tong, Wei Zhang

**Affiliations:** Department of Pharmacology, Pharmacy College, Nantong University, Nantong 226001, Jiangsu, China (Drs Jiang, Huang, Chen, Tong, and Zhang)

**Keywords:** brain-derived neurotrophic factor, chronic social defeat stress, depression, hippocampal neurogenesis, tetramethylpyrazine

## Abstract

**Background:**

Current antidepressants are clinically effective only after several weeks of administration. Tetramethylpyrazine (TMP) is an identified component of *Ligusticum wallichii* with neuroprotective effects. Here, we investigated the antidepressant effects of TMP in mice models of depression.

**Methods:**

Antidepressant effects of TMP were first detected in the forced swim test (FST) and tail suspension test (TST), and further assessed in the chronic social defeat stress (CSDS) model. Changes in the brain-derived neurotrophic factor (BDNF) signaling pathway and in hippocampal neurogenesis after CSDS and TMP treatment were then investigated. A tryptophan hydroxylase inhibitor and BDNF signaling inhibitors were also used to determine the mechanisms of TMP.

**Results:**

TMP exhibited potent antidepressant effects in the FST and TST without affecting locomotor activity. TMP also prevented the CSDS-induced symptoms. Moreover, TMP completely restored the CSDS-induced decrease of BDNF signaling pathway and hippocampal neurogenesis. Furthermore, a blockade of the BDNF signaling pathway prevented the antidepressant effects of TMP, while TMP produced no influence on the monoaminergic system.

**Conclusions:**

In conclusion, these data provide the first evidence that TMP has antidepressant effects, and this was mediated by promoting the BDNF signaling pathway.

## Introduction

Depression is a common but serious mental disorder that affects more than 15% of the population in the world, and this kind of neuropsychiatric disorder is usually associated with significant functional impairments and health care costs (Kessler et al., 1994; Olesen et al., 2012). Current antidepressants, like selective serotonin reuptake inhibitors (SSRIs), are the mainstay in the treatment for depression (Berton and Nestler, 2006). However, a lot of depressed patients do not respond well to presently available antidepressants and suffer from their severe side effects (McGrath et al., 2006). Therefore, it is necessary to explore new antidepressants with better efficacy and fewer side effects.

Recently, it has been widely accepted that brain-derived neurotrophic factor (BDNF) and cyclic Adenosine monophosphate (cAMP) response element-binding protein (CREB) play critical roles in the pathophysiology of depression (Krishnan and Nestler, 2008). CREB is an important transcription factor in the brain which controls the biosynthesis of many pro-survival proteins, including BDNF (Finkbeiner et al., 1997). BDNF also induces the phosphorylation and activation of CREB by combining the tyrosine kinase B (TrkB) receptor and promoting the MAPK-ERK (extracellular regulated protein kinase) and PI3K–protein kinase B (AKT) signaling pathways, two key downstream signaling pathways of BDNF (Shaywitz and Greenberg, 1999; Lim et al., 2008). Previous studies showed that the levels of BDNF and CREB in the hippocampus and medial prefrontal cortex (mPFC) are modulated by chronic stress and antidepressant treatment (Nibuya et al., 1996; Thome et al., 2000; Laifenfeld et al., 2005; Blendy, 2006; Gronli et al., 2006; Castren and Rantamaki, 2010; Razzoli et al., 2011; Opal et al., 2013; Zhou et al., 2013). Deficiency of the BDNF/CREB function made rodents susceptible to depression, while administration of BDNF/CREB produced antidepressant-like effects in models of depression (Chen et al., 2001; Shirayama et al., 2002; Hoshaw et al., 2005; Advani et al., 2009). This evidence indicates that stimulation of BDNF/CREB could provide a new approach to the treatment of depression.

Tetramethylpyrazine (TMP) is a purified chemical identified as a component of *Ligusticum wallichii*. By now, a wide range of neurotrophic and neuroprotective effects of TMP have been found, including protection against Parkinson’s disease and cerebral ischemia/reperfusion (Kao et al., 2006; Wang et al., 2007), protection of hippocampal neurons from excitotoxicity (Shih et al., 2002), improvement of memory (Wu et al., 2013), and promotion of the proliferation and differentiation of neural stem cells via increasing phosphorylation of phospho-extracellular regulated protein kinase 1/2 (pERK1/2) (Tian et al., 2010). A recent report showed that TMP also enhances the phosphorylation of hippocampal CREB (Wu et al., 2013). We thus speculated that TMP may have antidepressant effects. In this study, we assessed the antidepressant-like effects of TMP using various depression tests, including the forced swimming test (FST) and tail suspension test (TST), and the chronic social defeat stress (CSDS) model of depression. Furthermore, the molecular mechanisms for these effects were explored.

## Materials and methods

### Animals

Adult male C57BL/6J mice (8–10 weeks old) and CD1 mice (50 weeks old) were obtained from the Experimental Animal Center of the Medical Collegea at Nantong University. Before testing, mice were housed five per cage under standard conditions (12h light/dark cycle; lights on from 0700 to 1900h; 23±1°C ambient temperature; 55±10% relative humidity) for 1 week with free access to food and water. Each experimental group consisted of 12 mice. Behavioral experiments were carried out during the light phase. The experimental procedures involving animals and their care were conducted in compliance with the National Institutes of Health Guide for Care and Use of Laboratory Animals and with the European Communities Council Directive of 24 November 1986 (86/609/EEC).

### Materials

TMP (purity > 98%), 5-Bromo-2-deoxyUridine (Brdu), fluoxetine, and p-chlorophenylalanine methyl ester (PCPA) were purchased from Sigma. K252a was purchased from Alomone Laboratories. Chicken anti-BDNF neutralizing antibody and chicken Immunoglobulin Y (IgY) control Ig were purchased from Promega. The repeated vehicle/drug treatment of control and stressed mice was performed once daily at 0900–1100h. The dosages of TMP, Brdu, fluoxetine, K252a, anti-BDNF neutralizing antibody, and PCPA were chosen based on previous reports (Zhu et al., 2010, Jiang et al., 2012, Wu et al., 2013). TMP, Brdu, fluoxetine, and PCPA were administered i.p. in a volume of 10ml/kg. K252a, Chicken anti-BDNF neutralizing antibody, and chicken IgY control Ig were i.c.v. infused.

### Forced Swimming Test

The FST was performed in C57BL/6J mice according to our previous report (Jiang et al., 2012). Briefly, mice were individually placed into a glass cylinder (25cm in height, 10cm in diameter) filled with 20cm high water (25±1°C) for 30min after a single injection. The water was replaced after each trial. All mice were forced to swim for 6min, and the duration of immobility was recorded during the last 4min by an investigator blind to the study. Immobility time was defined as the time spent by the mouse floating in the water without struggling, and making only those movements necessary to keep its head above the water.

Additional experimental procedures are available online in Supplementary Material.

### Statistical Analysis

All analyses were performed using SPSS 13.0 software (SPSS Inc.) and data are presented as mean ± standard error of the mean (SEM). Differences between mean values were evaluated using one-way or two-way analyses of variance (ANOVA), as appropriate. For all one-way ANOVAs, post hoc tests were performed using the least significant difference (LSD) test. For all two-way ANOVAs, Bonferroni post hoc tests were used to assess isolated comparisons. A *p-*value < 0.05 was considered statistically significant.

## Results

### TMP Produces Antidepressant-Like Effects in the FST and TST

**Figure 1.**
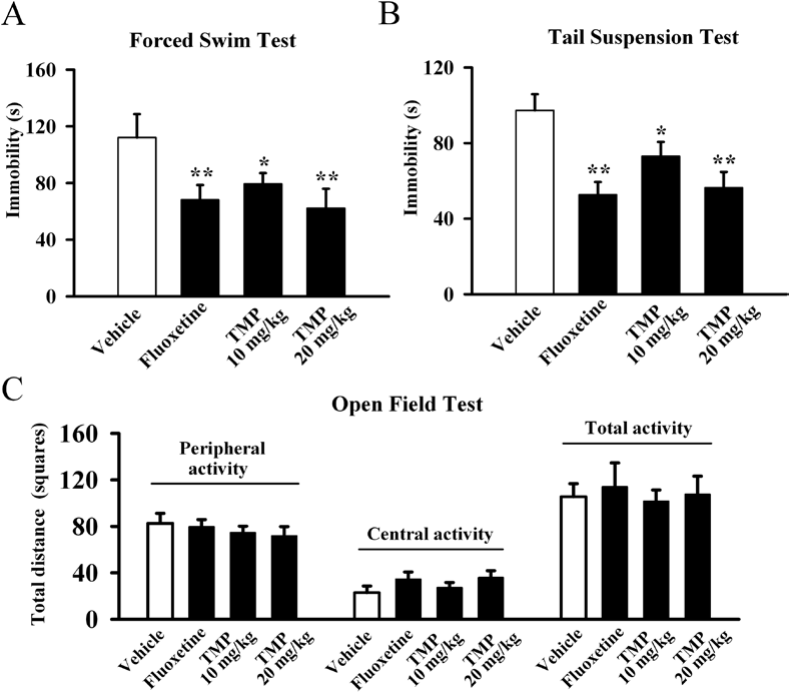
Tetramethylpyrazine (TMP) produces antidepressant-like effects in the forced swimming test (FST) and tail suspension test. C57BL/6J mice were i.p. injected with a single dose of vehicle (control, 3% DMSO), fluoxetine (20mg/kg), or TMP (10 or 20mg/kg). The behavioral tests were conducted 30min after the injection. TMP significantly decreased the immobility time of C57BL/6J mice in (A) the FST and (B) the TST. (C) TMP treatment produced no effects on the spontaneous locomotor activity in the open ﬁeld test. The data are expressed as mean ± standard error of the mean (n = 10); * *p* < 0.05, ** *p* < 0.01 vs. vehicle control by one-way analysis of variance followed by a post hoc least significant difference (LSD) test.

FST and TST are two widely-used behavioral tests for assessing potential antidepressant-like effects (Cryan and Holmes, 2005; Cryan and Slattery, 2007). We thus first examined the antidepressant effects of TMP in the FST. TMP (10 or 20mg/kg, dissolved in 3% DMSO) or fluoxetine (20mg/kg, used as a positive control, also dissolved in 3% DMSO) was administrated i.p. Data were subjected to a one-way ANOVA with drug treatment as the factor, which revealed a significant main effect of drug treatment [F(3, 36) = 28.357, *p* < 0.01]. Post hoc analysis showed that, compared to the control group, 10mg/kg TMP treatment induced a 30±4.1% decrease of immobility time in the FST and 20mg/kg TMP treatment induced a 44±3.1% decrease (Figure 1A). Similarly, fluoxetine also significantly reduced the immobility time (n = 10, *p* < 0.01 vs. control), consistent with previous reports (Holick et al., 2008)

We also performed the TST to assess the antidepressant-like effects of TMP (Figure 1B). A significant main effect of drug treatment [F(3, 36) = 33.112, *p* < 0.01] was revealed. Post hoc analysis indicated that, as in the FST, TMP robustly reduced the duration of immobility time in the TST at both 10mg/kg and 20mg/kg (n = 10, *p* < 0.01 vs. control). Fluoxetine also decreased immobility time as expected (n = 10, *p* < 0.01 vs. control).

Since there is a possibility that TMP produces effects on spontaneous locomotor activity, which may contribute to immobility in the FST and TST (Bourin et al., 2001), naive mice administrated TMP were exposed to the open-ﬁeld apparatus for 5min. We found no difference in the number of squares an animal crossed in the center area or the periphery area between all groups (Figure 1C), and ANOVA revealed no effects for drug treatment [F(3, 36) = 1.271, *p* = 0.298]. These data indicate that the TMP-induced decrease of immobility in the FST and TST was not due to locomotor hyperactivity.

### Chronic TMP Treatment Restores the CSDS-Induced Depressive Symptoms

**Figure 2.**
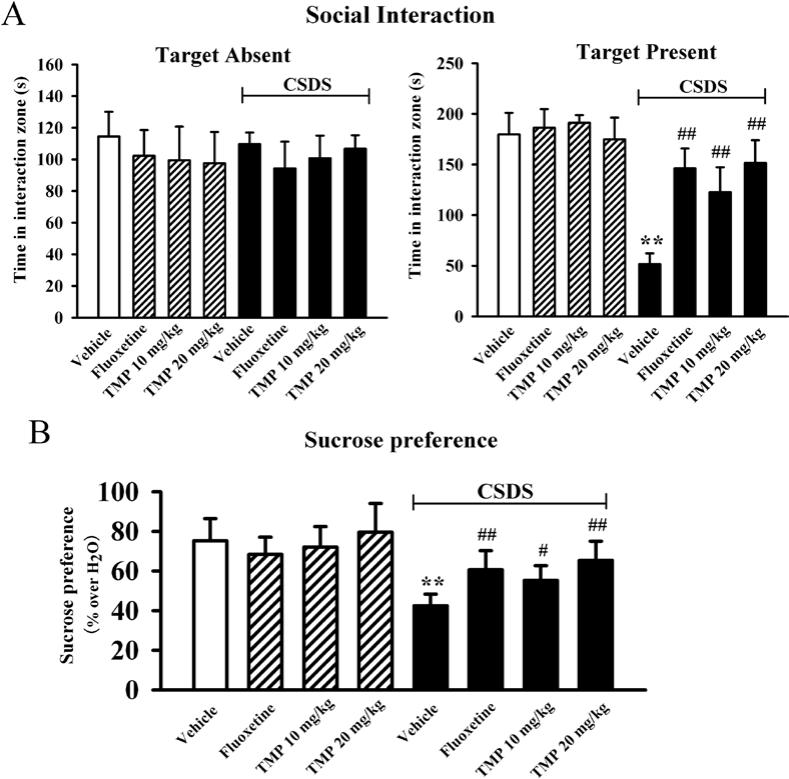
Tetramethylpyrazine (TMP) produces robust antidepressant effects in the chronic social defeat stress (CSDS) model of depression. C57BL/6J mice were exposed to defeat stress for 10 d and received a daily injection of vehicle, fluoxetine (20mg/kg), or TMP (10 or 20mg/kg) for another 14 d; behavioral tests were then conducted. (A) The antidepressant effects of TMP in the social interaction test. CSDS + TMP mice spent significantly more time engaged in social interaction than CSDS + vehicle mice. (B) TMP treatment reversed the decrease in sucrose consumption induced by CSDS. CSDS + TMP mice displayed a higher sucrose preference than CSDS + vehicle mice. Data are expressed as means ± standard error of the mean (n = 10); * *p* < 0.01 vs. vehicle control; ^#^ *p* < 0.05, ^##^ *p* < 0.01 vs. CSDS + vehicle group. Comparison was made by two-way analysis of variance followed by a post hoc Bonferroni’s test.

We further characterize the antidepressant effects of TMP in the CSDS model of depression, which mimics many symptoms of depression in human (Berton et al., 2006). We examined the effects of TMP on the social interaction and sucrose intake as indices of CSDS-induced responses. As shown in Figure 2A, while all mice spent similar amounts of time in the interaction zone in the absence of an aggressor, defeated mice spent about 71±4.9% less time in the interaction zone compared to control mice when an aggressor was introduced into the cage (n = 10, *p* < 0.01 vs. control), consistent with previous reports (Tsankova et al., 2006). Chronic TMP administration completely restored the CSDS-induced decrease of social interaction, especially at 20mg/kg (n = 10, *p* < 0.01 vs. CSDS), similar to fluoxetine. Data analysis also revealed a significant interaction [F(3, 72) = 68.242, *p* < 0.01], with significant effects for CSDS [F(1, 72) = 58.712, *p* < 0.01] and drug treatment [F(3, 72) = 18.445, *p* < 0.01].

The sucrose preference test was then performed, and Figure 2B illustrates the effects of CSDS and TMP on the sucrose intake. Two-way ANOVA reported a significant interaction [F(3, 72) = 18.563, *p* < 0.01], with significant effects for CSDS [F(1, 72) = 27.346, *p* < 0.01] and drug treatment [F(3, 72) = 9.244, *p* < 0.01]. We found that chronic defeat stress produced a 43±6.4% decrease in the sucrose consumption of C57BL/6J mice (n = 10, *p* < 0.01 vs. control). While TMP produced no significant effects in naive mice, 14 d treatment of TMP significantly increased the sucrose intake of stressed animals, also resulting in an antidepressant-like effect. Further analysis revealed that the sucrose intake was increased by 30±3.7% and 54±4.8% with administration of 10mg/kg and 20mg/kg TMP (n = 10, *p* < 0.01 vs. CSDS), respectively.

In a parallel series, CSDS-stressed mice were injected with a single dose of TMP to see if TMP has acute antidepressant effects. However, acute TMP treatment could not reverse the CSDS-induced decrease of social interaction (n = 10, Supplementary Figure 1A), and ANOVA revealed a significant effect for CSDS [F(1, 54) = 39.642, *p* < 0.01] but no effect for TMP [F(2, 54) = 0.223, *p* = 0.646]. Similarly, acute TMP injection did not increase the sucrose intake of stressed mice (n = 10, Supplementary Figure 1B), and ANOVA also revealed a significant effect for CSDS [F(1, 54) = 20.492, *p* < 0.01] but no effect for TMP [F(2, 54) = 0.319, *p* = 0.591].

### The CSDS-Induced Decrease in Hippocampal Neurogenesis was Restored by TMP

**Figure 3.**
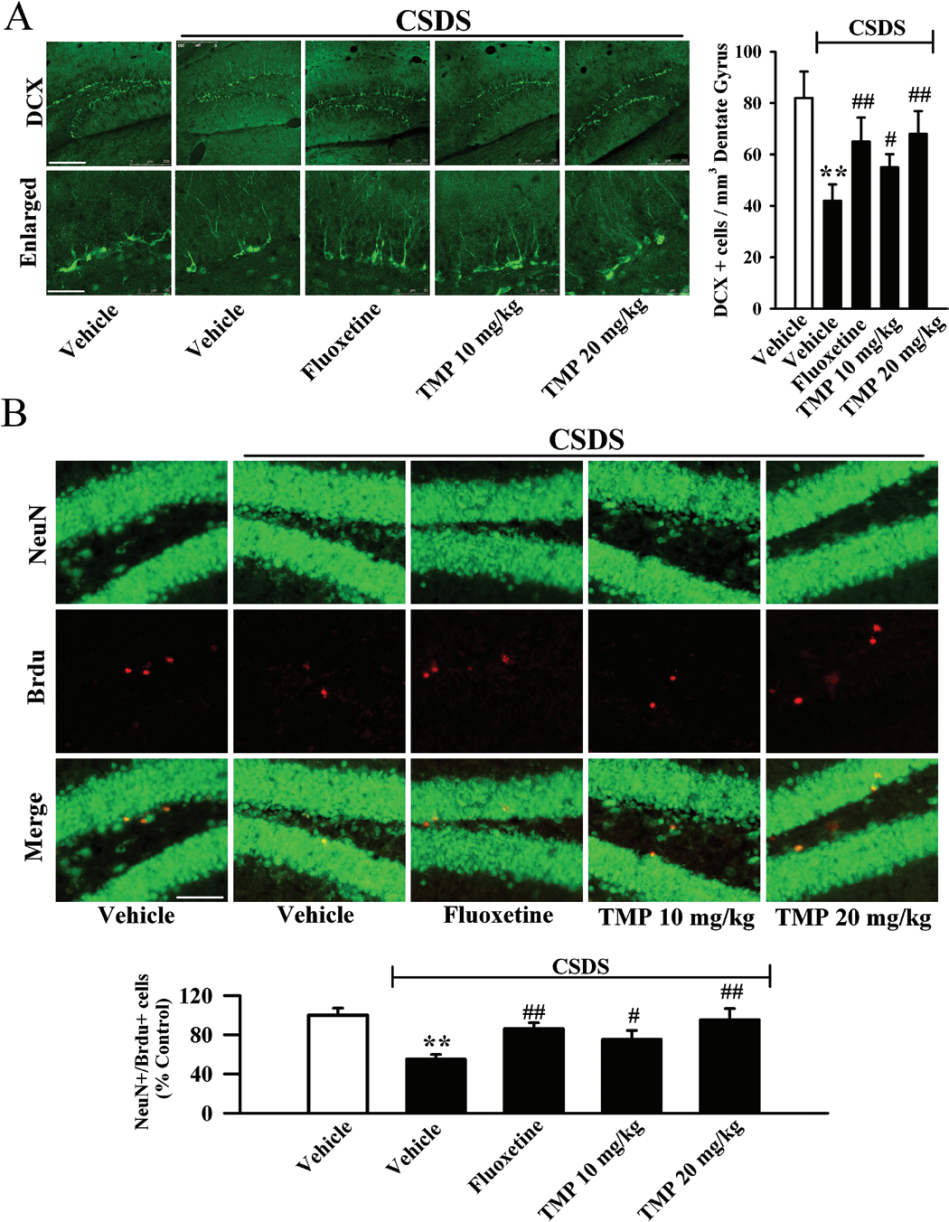
Tetramethylpyrazine (TMP) administration restores the decreased hippocamal neurogenesis caused by defeat stress. (A) Representative confocal microscopic images showed the localization of doublecortin (DCX; green) in the dentate gyrus (DG). The scale bar is 200 µm for representative images and 50 µm for enlarged images, respectively. Density statistics showed that chronic TMP treatment significantly increased the number of DCX-stained cells in the DG of chronic social defeat stress (CSDS) animals. (B) Representative microscopic images showed the co-staining (yellow) of Neuronal Nuclei (NeuN) (green) and 5-Bromo-2-deoxyUridine (Brdu) (red) in the DG. The majority of Brdu+ cells are doubly labeled with the neuronal marker NeuN and located within the granule cell layer. The scale bar is 100 µm. Density statistics showed that TMP administration completely reversed the CSDS-induced decrease of NeuN+/Brdu+ cells number in the DG. Data are expressed as means ± standard error of the mean (n = 5); * *p* < 0.01 vs. vehicle control; ^#^ *p* < 0.05, ^##^ *p* < 0.01 vs. CSDS + vehicle group. Comparison was made by two-way analysis of variance followed by a post hoc Bonferroni’s test.

It has been demonstrated that chronic stress significantly reduces cell proliferation in the dentate gyrus (DG) region (Lagace et al., 2010), and that hippocampal neurogenesis is required for the beneficial effects of common antidepressants like fluoxetine (Santarelli et al., 2003). We thus examined whether TMP can prevent the stress-induced effects on neurogenesis. Here, hippocampal proliferation was studied by doublecortin (DCX) immunohistochemistry in the DG region, as we previously described (Jiang et al., 2012). Figure 3A shows the immunohistochemical staining of DCX+ cells. Two-way ANOVA indicated a significant interaction [F(3, 17) = 40.675, *p* < 0.01], with significant effects for CSDS [F(1, 17) = 116.451, *p* < 0.01] and drug treatment [F(3, 17) = 28.327, *p* < 0.01]. We found that CSDS resulted in a 57±7.8% reduction in the number of DCX+ cells (n = 5, *p* < 0.01 vs. control), and that chronic TMP treatment at 20mg/kg completely restored this change, similar to fluoxetine (n = 5, *p* < 0.01 vs. glyceraldehyde-3-phosphate dehydrogenase (CSDS); Figure 3A).

Newly-generated cells in the DG differentiate into neurons within 28 d after their birth (Kempermann et al., 2003). To determine whether the newborn cells induced by TMP treatment differentiated into new neurons, Brdu was administrated and Neuronal Nuclei (NeuN) was employed as a marker for mature neurons (Mullen et al., 1992). Figure 3B shows that CSDS resulted in a 45±6.3% reduction in the number of NeuN+/Brdu+ co-labeling cells in the DG (n = 5, *p* < 0.01 vs. control), and TMP treatment fully reversed this change (n = 5, *p* < 0.01 vs. CSDS), suggesting that the TMP-induced newborn cells in the DG preferentially differentiated into neurons. Two-way ANOVA indicated a significant interaction [F(3, 17) = 24.871, *p* < 0.01], with significant effects for CSDS [F(1, 17) = 93.612, *p* < 0.01] and drug treatment [F(3, 17) = 18.113, *p* < 0.01]. These data indicate that TMP could resverse the CSDS-induced decrease in hippocampal neurogenesis.

### TMP Treatment Reverses the CSDS-Induced Decrease in BDNF Signaling Pathway

**Figure 4.**
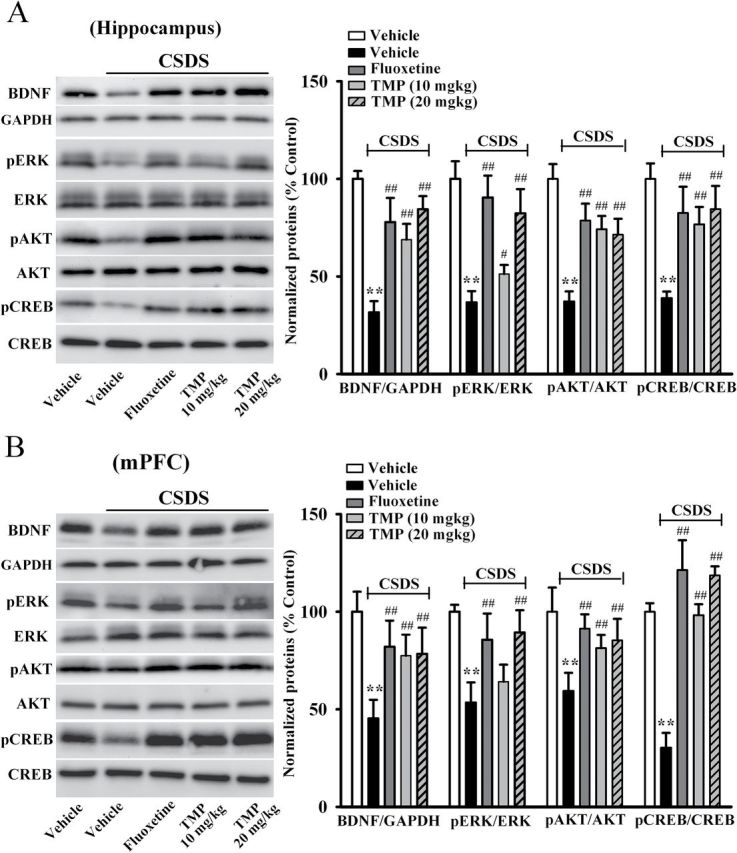
Tetramethylpyrazine (TMP) treatment reverses the chronic social defeat stress (CSDS)-induced decrease of the brain-derived neurotrophic factor (BDNF) signaling pathway. (A) Western blotting results showed that TMP treatment restored the CSDS-induced decrease of BDNF, phospho-extracellular regulated protein kinase 1/2 (pERK1/2), the active form of protein kinase B (pAKT), and phospho-cAMP response element binding protein (pCREB) protein levels in the hippocampus. CSDS + TMP mice displayed significantly higher hippocampal expression of BDNF, pERK1/2, pAKT and pCREB than CSDS + vehicle mice. (B) As in the hippocampus, TMP administration also restored the CSDS-induced inhibition of BDNF, pERK1/2, pAKT, and pCREB levels in the medial prefrontal cortex (mPFC). CSDS + TMP mice displayed higher BDNF, pERK1/2, pAKT, and pCREB expression in the mPFC, compared to CSDS + vehicle mice. Data are expressed as means ± standard error of the mean (n = 5); * *p* < 0.01 vs. vehicle control; ^#^ *p* < 0.05, ^##^ *p* < 0.01 vs. CSDS + vehicle group. Comparison was made by two-way analysis of variance followed by a post hoc Bonferroni’s test. AKT, PI3K–protein kinase B; CREB, cyclic Adenosine monophosphate (cAMP) response element-binding protein; ERK, extracellular regulated protein kinase; glyceraldehyde-3-phosphate dehydrogenase (GAPDH).

The brain derived neurotrophic factor-extracellular regulated protein kinase (BDNF-ERK/AKT-CREB) signaling cascade plays an important role in the pathophysiology of depression, and is critical for hippocampal neurogenesis (Gourley et al., 2008; Lee and Son, 2009; Castren and Rantamaki, 2010). Therefore, we performed Western blotting to measure the BDNF protein level in the hippocampus and mPFC following CSDS and TMP treatment. The BDNF protein level was expressed as a ratio of the expression of GAPDH. As shown in Figure 4A, CSDS robustly reduced BDNF expression in the hippocampus (n = 5, *p* < 0.01 vs. control), and 14 d treatment of TMP increased the BDNF protein level by 117±10.6% and 167±11.8% at dosages of 10mg/kg and 20mg/kg, respectively, similar to 20mg/kg fluoxetine (n = 5, *p* < 0.01 vs. CSDS). ANOVA also indicated a significant interaction [F(3, 17) = 38.912, *p* < 0.01], with significant effects for CSDS [F(1, 17) = 107.314, *p* < 0.01] and drug treatment [F(3, 17) = 27.534, *p* < 0.01]. In parallel to the hippocampus, CSDS decreased the BDNF expression in the mPFC (n = 5, *p* < 0.01 vs. control), and TMP treatment increased the BDNF protein level by 71±6.6% and 73±4.5% at dosages of 10mg/kg and 20mg/kg, respectively (n = 5, *p* < 0.01 vs. CSDS; Figure 4B). ANOVA also indicated a significant interaction [F(3, 17) = 22.432, *p* < 0.01], with significant effects for CSDS [F(1, 17) = 87.923, *p* < 0.01] and drug treatment [F(3, 17) = 17.612, *p* < 0.01].

Since the MAPK-ERK and PI3K-AKT pathways are two key downstream signaling pathways of BDNF (Lim et al., 2008), we then examined the levels of pERK1/2 (the active form of ERK1/2) and pAKT (the active form of AKT) in the hippocampus and mPFC, respectively. As shown in Figure 4A, chronic TMP treatment significantly reversed the CSDS-induced decrease in hippocampal pERK1/2 and pAKT expression (n = 5, *p* < 0.01 vs. CSDS), with ANOVA indicating a significant interaction [F(3, 17) = 43.543, *p* < 0.01] and significant effects for CSDS [F(1, 17) = 131.411, *p* < 0.01] and drug treatment [F(3, 17) = 36.727, *p* < 0.01]. Similarly, the decreased pERK1/2 and pAKT levels in the mPFC of stressed mice were also restored by TMP exposure (n = 5, *p* < 0.01 vs. CSDS; Figure 4B), with ANOVA indicating a significant interaction [F(3, 17) = 31.456, *p* < 0.01] and significant effects for CSDS [F(1, 17) = 98.787, *p* < 0.01] and drug treatment [F(3, 17) = 20.651, *p* < 0.01]. By contrast, the total ERK1/2 and AKT levels were unchanged among all treatment groups.

CREB is not only the nuclear downstream signaling molecule of BDNF, but also the transcription factor of BDNF protein (Finkbeiner et al., 1997; Shaywitz and Greenberg, 1999). Here, we found that CSDS significantly decreased the hippocampal phospho-cAMP response element binding protein (pCREB) level (n = 5, *p* < 0.01 vs. control), which was reversed by fluoxetine (n = 5, *p* < 0.01 vs. CSDS; Figure 4A). Moreover, TMP administration increased the hippocampal pCREB level of stressed mice, especially at 20mg/kg (n = 5, *p* < 0.01 vs. CSDS; Figure 4A). ANOVA indicated significant effects of CSDS [F(1, 17) = 112.322, *p* < 0.01] and drug treatment [F(3, 17) = 17.655, *p* < 0.01], as well as significant interaction between the two [F(3, 17) = 30.914, *p* < 0.01]. We also found that CSDS decreased the pCREB expression in the mPFC (n = 5, *p* < 0.01 vs. control), and this was completely restored by chronic TMP treatment (n = 5, *p* < 0.01 vs. CSDS; Figure 4B). ANOVA also indicated a significant interaction [F(3, 17) = 15.613, *p* < 0.01], with significant effects for CSDS [F(1, 17) = 76.454, *p* < 0.01] and drug treatment [F(3, 17) = 12.823, *p* < 0.01]. The level of total CREB protein was not altered. Together, the TMP-induced effects on the BDNF signaling pathway may be involved in its antidepressant effects.

### TMP Produced Antidepressant Effects Through BDNF Signaling Pathway

**Figure 5.**
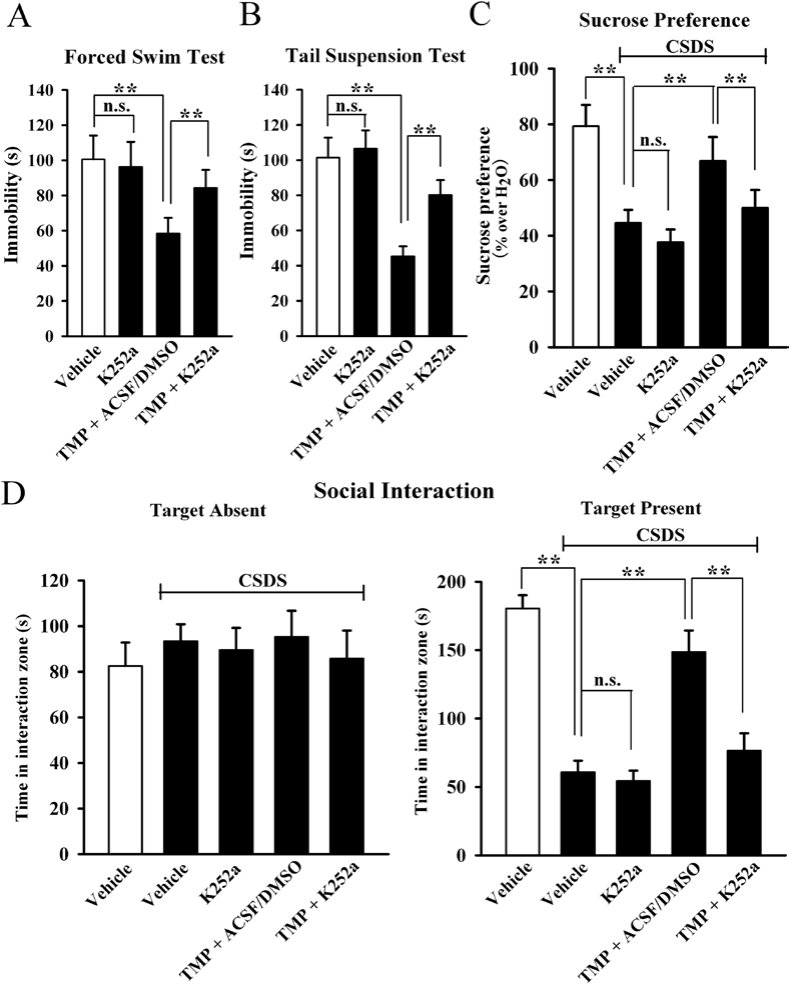
Blockade of brain-derived neurotrophic factor signaling cascade by K252a prevents the antidepressant actions of tetramethylpyrazine (TMP). K252a pretreatment before TMP administration dramatically prevented the TMP-induced decrease in the duration of immobility in the (A) forced swimming test and (B) in the tail suspension test. (C) Chronic social defeat stress (CSDS)-treated mice were co-treated with TMP and K252a for 14 d. CSDS + TMP + K252a mice displayed significantly lower sucrose consumption than CSDS + TMP + artificial cerebrospinal fluid-dimethyl sulphoxide (ACSF/DMSO) mice. (D) Co-treatment of TMP with K252a blocked the antidepressant effect of TMP in the social interaction test. CSDS + TMP + K252a mice displayed significantly lower social interaction than CSDS + TMP + ACSF/DMSO mice. Results are expressed as means ± standard error of the mean (n = 10); * *p* < 0.01; n.s., no significance. Comparison was made by one-way analysis of variance followed by a post hoc LSD test.

To further determine whether the BDNF system is necessary for the effects of TMP, the potent pharmacological inhibitor of BDNF receptor TrkB, K252a, was used (Tapley et al., 1992; Yan et al., 2010). Mice were first infused with K252a for 3 d, then treated with TMP (20mg/kg) and placed in the FST. K252a pretreatment dramatically prevented the TMP-induced decrease of immobility in the FST (Figure 5A). We also found that K252a pretreatment significantly prevented the TMP-induced effects in the TST (Figure 5B). Moreover, CSDS-treated mice were co-treated with TMP (20mg/kg) and K252a for 14 d, and behavioral tests were then performed. As shown in Figure 5D, co-treatment of TMP with K252a inhibited the social interaction in the CSDS-treated mice. Also, co-treatment of TMP with K252a inhibited the sucrose preference in the CSDS-treated mice (Figure 5C).

**Figure 6.**
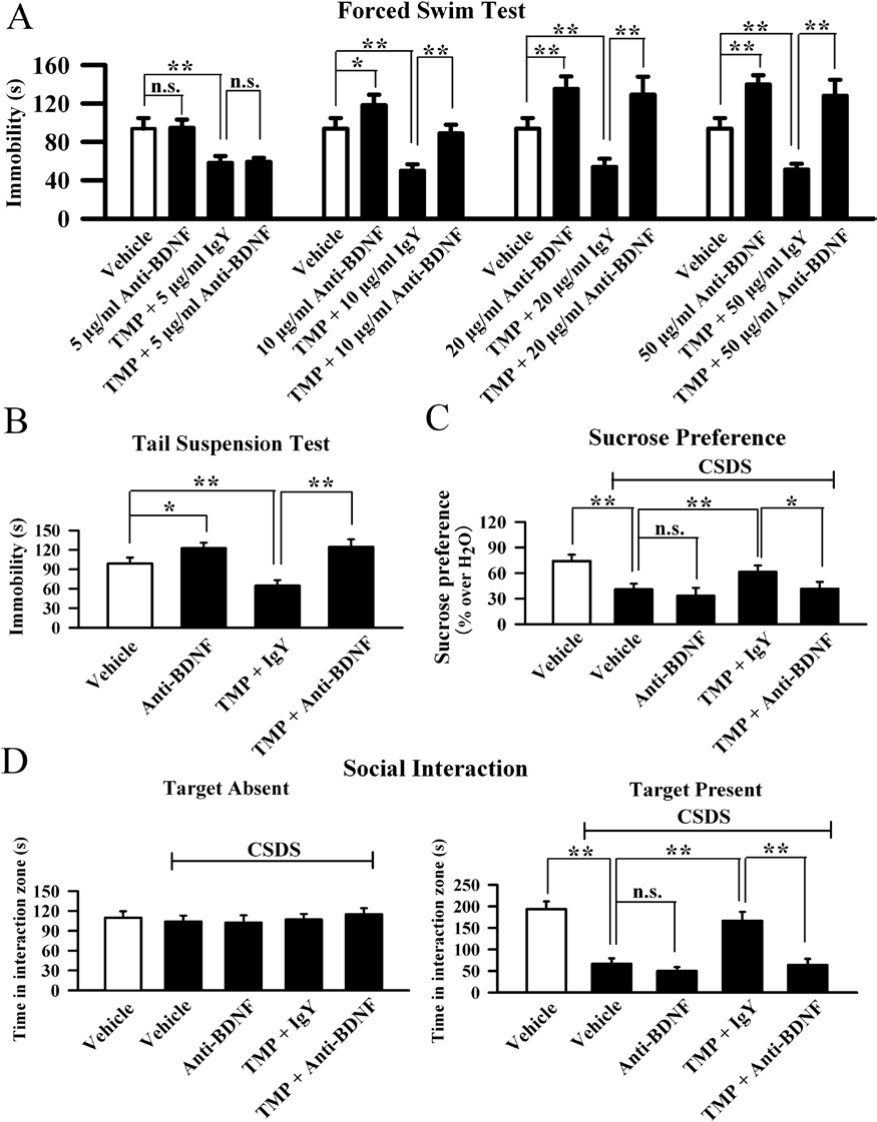
Blockade of the brain-derived neurotrophic factor (BDNF) signaling cascade by anti-BDNF infusion abolishes the antidepressant effects of tetramethylpyrazine (TMP). (A) Pre-infusion of an anti-BDNF antibody blocked the TMP-induced decrease of immobility in the forced swimming test in a dose-dependent manner between 5 and 20 µg/ml. (B) Pre-infusion of the anti-BDNF antibody (20 µg/ml) also prevented the TMP-induced decrease of immobility in the tail suspension test. (C) Chronic social defeat stress (CSDS)-treated mice were co-treated with TMP and anti-BDNF antibody (20 µg/ml) for 14 d. CSDS + TMP + anti-BDNF mice displayed significantly lower sucrose consumption than CSDS + TMP + Immunoglobulin Y (IgY) mice. (D) Co-treatment of TMP with the anti-BDNF antibody (20 µg/ml) blocked the antidepressant effect of TMP in the social interaction test. CSDS + TMP + anti-BDNF mice displayed significantly lower social interaction than CSDS + TMP + IgY mice. Results are expressed as means ± standard error of the mean (n = 10); * *p* < 0.05, ** *p* < 0.01; n.s., no significance. Comparison was made by one-way analysis of variance followed by a post hoc LSD test.

In a parallel series, we used an anti-BDNF antibody to specifically block the BDNF system. Mice were first infused with the anti-BDNF antibody for 3 d, then treated with TMP (20mg/kg) followed by behavioral tests. As shown in Figure 6A, anti-BDNF infusion alone increased the immobility time of C57BL/6J mice in the FST (consistent with previous studies showing that deficiency of BDNF made rodents susceptible to stress; Advani et al., 2009), and IgY had no noticeable effects on TMP’s shortening of the immobility time. However, anti-BDNF infusion prevented the effects of TMP in the FST in a dose-dependent manner between 5 and 20 µg/ml. Similarly, 20 µg/ml anti-BDNF infusion also fully blocked the antidepressant effects of TMP in the TST (Figure 6B). Moreover, CSDS-treated mice were co-treated with TMP (20mg/kg) and anti-BDNF antibody (20 µg/ml) for 14 d, and behavioral tests were then performed. As shown in Figure 6D, anti-BDNF infusion abolished the antidepressant effects of TMP in the social interaction test. Also, anti-BDNF infusion abolished the antidepressant effects of TMP in the sucrose preference test (Figure 6C).

**Figure 7.**
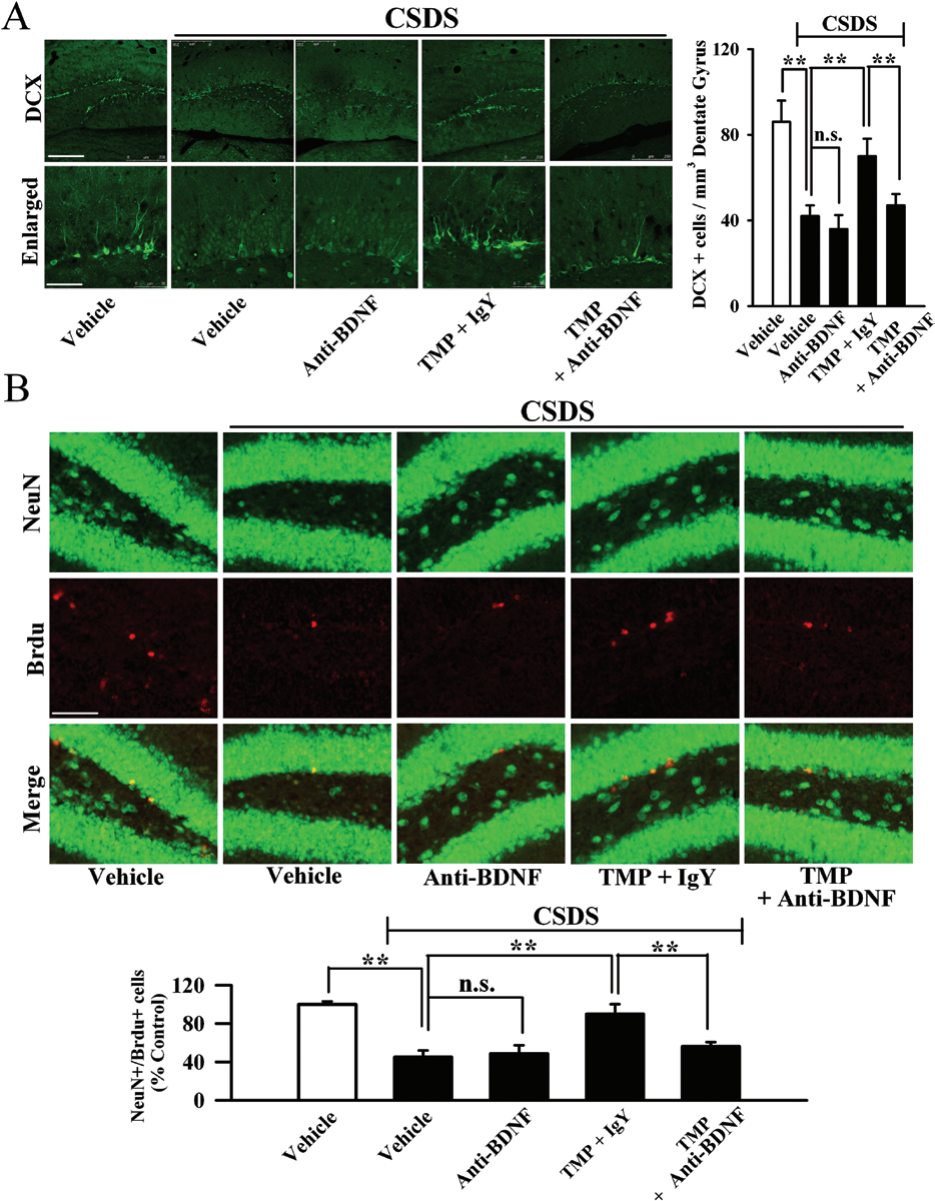
Anti-BDNF (brain-derived neurotrophic factor) infusion blocks the neurogenic effects of tetramethylpyrazine (TMP). (A) Representative confocal microscopic images show the staining of doublecortin (DCX; green) in the dentate granule cell layer. The scale bar is 200 µm for representative images and 50 µm for enlarged images, respectively. Density statistics showed that the increased number of DCX positive cells induced by TMP was blocked by anti-BDNF infusion. (B) Representative microscopic images showed the co-staining (yellow) of Neuronal Nuclei (NeuN) (green) and 5-Bromo-2-deoxyUridine (Brdu) (red) in the dentate gyrus (DG). The scale bar is 100 µm. Density statistics showed that co-injection with the anti-BDNF antibody blocked the TMP effects on the amount of NeuN+/Brdu+ cells in the DG. Data are expressed as means ± standard error of the mean (n = 5); * *p* < 0.01; n.s., no significance. Comparison was made by one-way analysis of variance followed by a post hoc LSD test. CSDS, chronic social defeat stress; Immunoglobulin Y (IgY).

**Figure 8.**
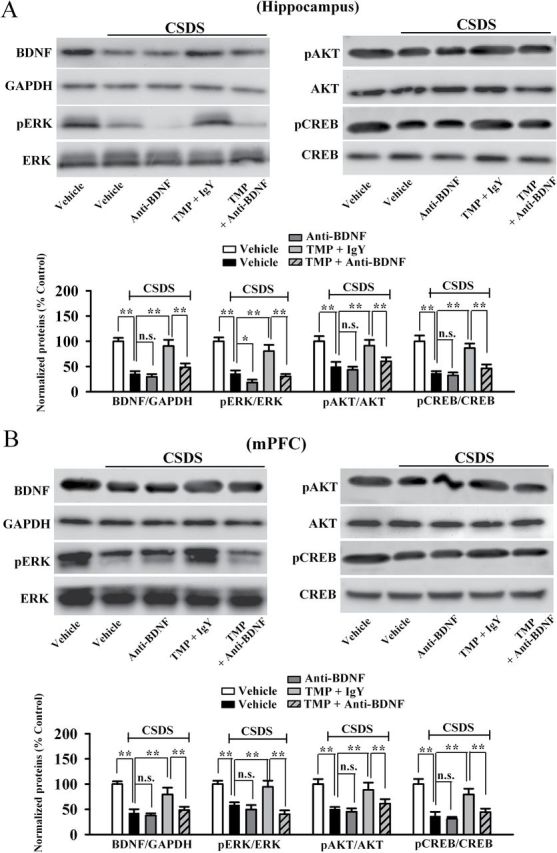
Anti-BDNF (brain-derived neurotrophic factor) infusion also prevents the effects of tetramethylpyrazine (TMP) on the BDNF signaling pathway. (A) Western blotting results showed that the enhancement of BDNF, phospho-extracellular regulated protein kinase 1/2 (pERK1/2), the active form of protein kinase B (pAKT), and phospho-cAMP response element binding protein (pCREB) expression in the hippocampus induced by TMP treatment was abolished by anti-BDNF infusion. (B) As in the hippocampus, Western blotting results indicated that the TMP-induced promotion of BDNF, pERK1/2, pAKT, and pCREB expression in the medial prefrontal cortex was also blocked by anti-BDNF antibody. Data are expressed as means ± standard error of the mean (n = 5); *p < 0.05, ** *p* < 0.01; n.s., no significance. Comparison was made by one-way analysis of variance followed by a post hoc LSD test. AKT, PI3K–protein kinase B; CREB, cyclic Adenosine monophosphate (cAMP) response element-binding protein; CSDS, chronic social defeat stress; ERK, extracellular regulated protein kinase; glyceraldehyde-3-phosphate dehydrogenase (GAPDH).

Next, we examined whether anti-BDNF infusion blocked the effects of TMP on hippocampal neurogenesis and the BDNF-ERK/AKT-CREB signaling cascade. There was a significant difference between control and CSDS groups. More importantly, in parallel to the behavioral data, anti-BDNF infusion significantly blocked the effects of TMP on neurogenesis (Figure 7A and 7B), BDNF (Figure 8A), pERK1/2 (Figure 8A), pAKT (Figure 8A), and pCREB (Figure 8A) levels in the hippocampus. Similarly, anti-BDNF infusion also prevented the effects of TMP on BDNF (Figure 8B), pERK1/2 (Figure 8B), pAKT (Figure 8B), and pCREB (Figure 8B) levels in the mPFC region. Together, these data indicate that the antidepressant effects of TMP require the BDNF system.

### Serotonin Depletion Does Not Alter the Antidepressant Effects of TMP

Given that monoamine systems are also involved in the pathophysiology of depression (Sulser et al., 1962; Berton and Nestler, 2006) and that SSRIs are the most widely used antidepressants, we further tested whether the TMP-induced antidepressant effects depended on the serotonin signaling. The tryptophan hydroxylase inhibitor PCPA was used to deplete serotonin. Although PCPA does not increase depression-like behavior, it blocks the antidepressant effects of fluoxetine (Heurteaux et al., 2006; Coryell et al., 2009). Mice were given i.p. injection of PCPA (300mg/kg, daily, 3 d) following injection of TMP (20mg/kg), and then antidepressant activity was assessed. Our data show that PCPA had no influence on the TMP-induced effects in the FST (Supplementary Figure 2A). Also, PCPA produced no influence on the TMP-induced effects in the TST (Supplementary Figure 2B).

To further validate this, stressed mice were co-injected with TMP (20mg/kg) and PCPA for 14 d, and then tested for sucrose preference and social interaction. Supplementary Figure 2C and D show that PCPA had no effects on either the sucrose consumption or social interactions of CSDS + TMP–treated mice. These results suggest that TMP produces antidepressant effects through mechanisms different from fluoxetine.

## Discussion

The major findings of this study are as follows. First, TMP produces antidepressant effects in multiple animal models screening for antidepressant activity, including the CSDS paradigm, FST, and TST. Second, the antidepressant effects of TMP require the BDNF signaling pathway, since they could be prevented by selective inhibition of BDNF-TrkB signaling in the brain. Together, these data indicate that TMP could be developed as a novel antidepressant.

The compound TMP was selected in our study by virtue of the knowledge that it could enhance the phosphorylation of hippocampal CREB, since CREB is closely correlated with the pathophysiology of depression (Gass and Riva, 2007; Wu et al., 2013). Besides, previous studies showed that TMP could protect hippocampal neurons from excitotoxicity, promote neurogenesis, and improve learning and memory (Shih et al., 2002; Tian et al., 2010; Wu et al., 2013). These findings also imply that TMP may possess antidepressant effects, since depression is accompanied by increased hippocampal neuronal death, decreased hippocampal neurogenesis, and also decreased learning and memory (Mizoguchi et al., 1992; Conrad, 2010; Lagace et al., 2010). Tsai and Liang (2001) demonstrated that TMP effectively penetrated the blood-brain barrier and could be enriched in the brain. Here, we first assessed the effects of TMP using the FST and TST, since the two tests have high predictive validity for detecting antidepressant activity (Cryan and Holmes, 2005; Cryan and Slattery, 2007). We found that acute injection of 20mg/kg TMP produced a signiﬁcant reduction of immobility time in both the FST and TST. Moreover, TMP treatment had no influence on the locomotor activity of mice, indicating that the TMP-induced reduction of immobility was not due to locomotor abnormality. We further used the CSDS model to validate the effects of TMP, as the CSDS model has good predictive validity the symptomatology of stress-related disorders like depression (Berton et al., 2006). Importantly, consecutive injection of TMP for 14 d significantly ameliorated the behavioral deficits of CSDS-treated mice to the basal level of non-stressed control mice, suggesting that TMP can be developed as a novel antidepressant.

It is well known that hippocampal neurogenesis can be modulated by common antidepressants, like fluoxetine (Perera et al., 2011). Our results showed that, similar to fluoxetine, TMP administration also restores the decreased hippocampal neurogenesis caused by CSDS, indicating that TMP may also be developed as a pro-neurogenic compound. The immunohistochemical results may be supported by previous reports showing that TMP promotes the proliferation and differentiation of neural stem cells into neurons, and also enhances neurogenesis in adult rat brains after focal ischemia (Tian et al., 2010). There are a lot of proteins that control neurogenesis. We finally selected BDNF in the study, as: (1) BDNF controls both hippocampal neurogeneis and the pathogenesis of depression (Lee and Son, 2009; Castren and Rantamaki, 2010); (2) the biosynthesis of BDNF is regulated by CREB, which has already been demonstrated to be affected by TMP (Conti et al., 2002; Wu et al., 2013); (3) a previous study showed that TMP treatment promotes the expression of BDNF after severe brain injury in rats (Ma et al., 2008); and (4) TMP also increases the phosphorylation of pERK1/2, a key downstream kinase of BDNF (Tian et al., 2010). Here, we found that CSDS stress robustly decreased the BDNF expression in both the hippocampus and mPFC, two regions closely implicated in the pathogenesis of depression (Pittenger and Duman, 2008; Li et al., 2010). These data are consistent with previous reports (Smith et al., 1995; Duman and Monteggia, 2006; Castren and Rantamaki, 2010), proving the effectiveness of our depression model. As we expected, TMP treatment significantly reversed the CSDS-induced decrease of BDNF protein, consistent with Ma et al.’s study (2008), which showed that TMP affects BDNF expression. Similarly, TMP also increased the BDNF downstream signaling molecules (pERK1/2, pAKT, and pCREB) of stressed mice, consistent with Tian et al. (2010) and Wu et al.’s (2013) results, which showed that TMP could enhance pERK1/2 and pCREB, respectively. There is also other evidence indicating that TMP could affect the BDNF system. For example, Wu et al. (2013) found that administration of TMP effectively reverses the scopolamine-induced deficits of memory, while BDNF plays a critical role in learning and memory (Yamada et al., 2002). Furthermore, the usage of either K252a or anti-BDNF antibody fully abolished the antidepressant effects of TMP, indicating that BDNF is involved in this response. The conclusion that TMP-induced antidepressant effects require the BDNF system should be reliable and believable, as the chicken anti-BDNF antibody used in this study has already been shown to be neutralizing and specific for BDNF (Chen et al., 2005; Zhu et al., 2010).

Since TMP produces antidepressant effects similar to fluoxetine, we need to consider the possibility that these effects may be also mediated through monoaminergic systems, especially the seretonergic system. Depleting serotonin by PCPA did nothing to influence the antidepressant action of TMP, while blockade of the BDNF-TrkB system prevented the effects of TMP, indicating that the antidepressant mechanism of TMP is distinct from those conventional antidepressants.

Collectively, TMP has wide-ranging biological effects, and many reveal positive therapeutic indices. Our results show that TMP possesses antidepressant-like properties through promotion of the BDNF signaling pathway, providing a new insight to understanding the pharmacological effects of TMP. More importantly, this study shed light on the development of new antidepressants with higher efficacy and fewer side effects. In addition to depression, the BDNF system is implicated in some other neuronal dysfunctions, like Alzheimer’s disease (Voineskos et al., 2011), so it is possible that TMP may also produce effects in these disorders, and this needs further study.

## Supplementary Material

For supplementary material accompanying this paper, visit http://www.ijnp.oxfordjournals.org/. The supplementary materials include experimental procedures and one figure

## Statement of Interest

The authors declare no conflict of interest.

## Supplementary Material

Supplementary Figure 1A
