# Supplementary figures and images for: Tetramethylpyrazine Produces Antidepressant-Like Effects in Mice Through Promotion of BDNF Signaling Pathway
Source: Int J Neuropsychopharmacol. 2015 Mar 6;18(8):pyv010. doi: 10.1093/ijnp/pyv010 (PMC4571627; doi:10.1093/ijnp/pyv010)

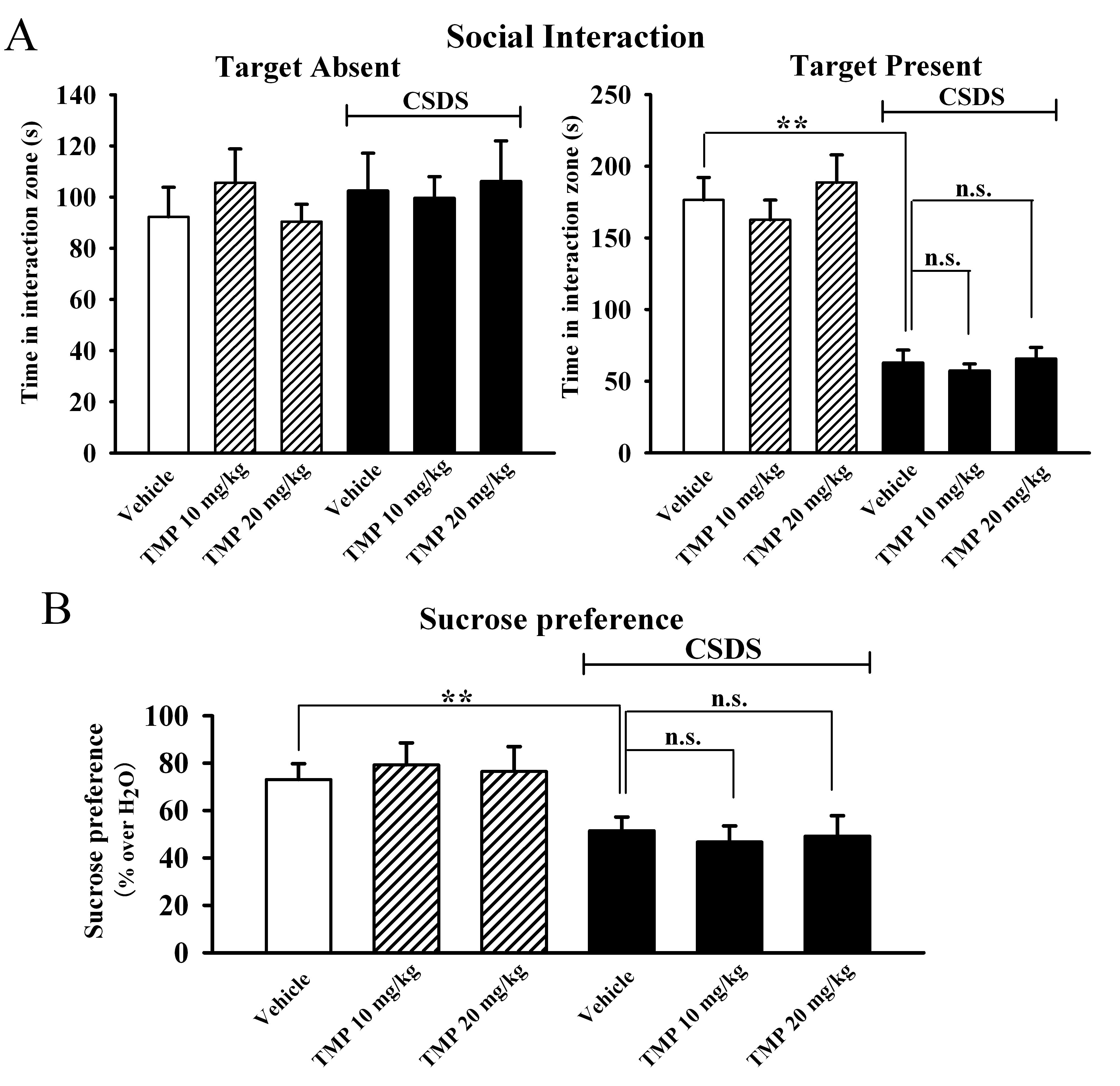

Supplement: Supplementary Figure 1A [file Figure_S1.tif]

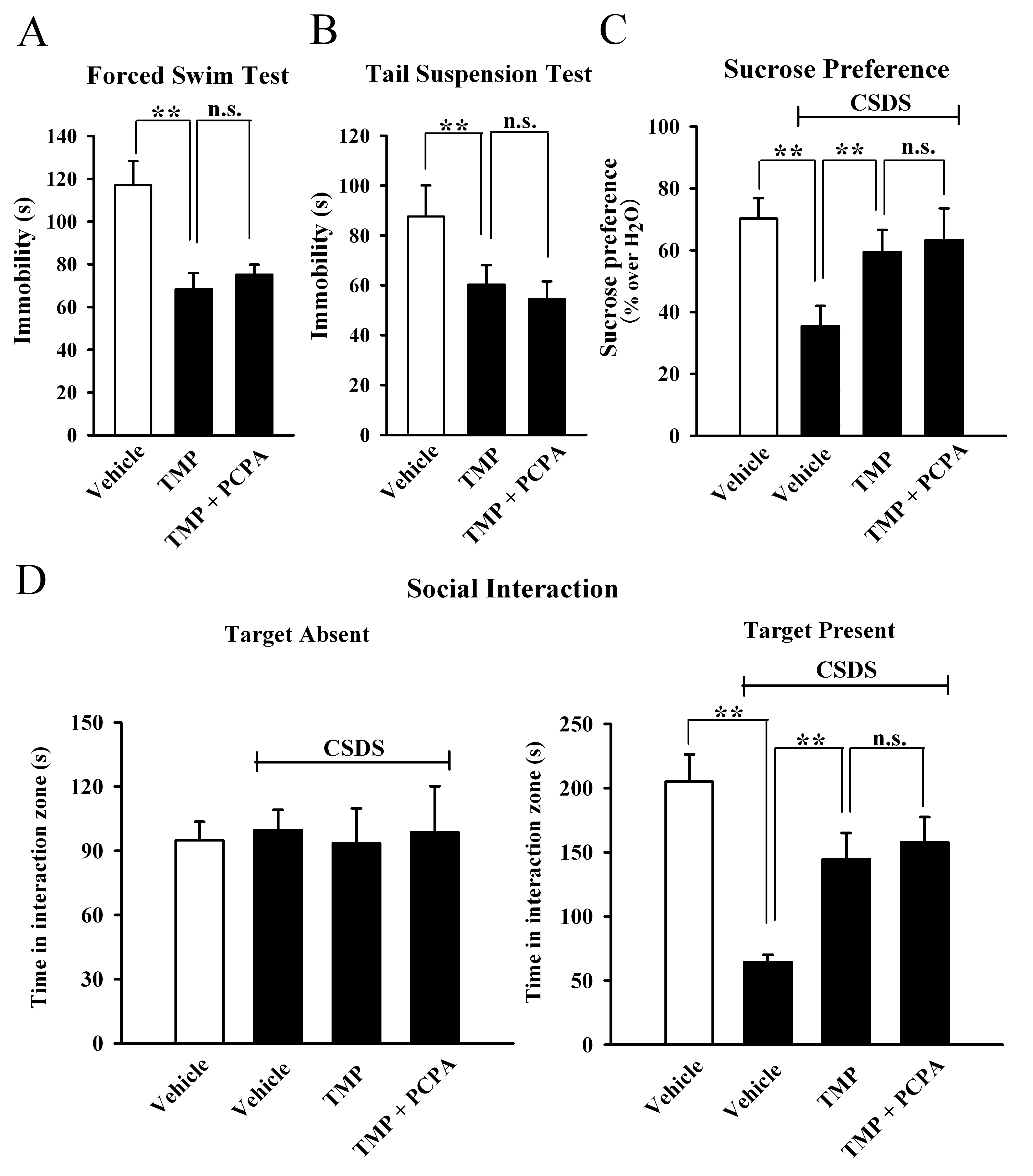

Supplement: Supplementary Figure 1A [file Figure_S2.tif]
