# Supplementary material for: Tetramethylpyrazine Produces Antidepressant-Like Effects in Mice Through Promotion of BDNF Signaling Pathway
Source: Int J Neuropsychopharmacol. 2015 Mar 6;18(8):pyv010. doi: 10.1093/ijnp/pyv010 (PMC4571627; doi:10.1093/ijnp/pyv010)
Supplement: Supplementary Figure 1A [file Revised_Supplemental_material.docx]

**Supplementary Information**

**Tetramethylpyrazine produces antidepressant-like effects in mice through promotion of BDNF signaling pathway**

Bo Jiang ^1, #^, Chao Huang ^1, #^, Xiang-Fan Chen ^1^, Li-Juan Tong ^1^ and Wei Zhang ^1, *^

^1^Department of Pharmacology, Pharmacy College, Nantong University, Nantong 226001, Jiangsu, China

**Running title:** Tetramethylpyrazine has antidepressant effects

**Category:** Regular research article

**Word count:** 5364 (main manuscript); 182 (abstract)

**Number of references:** 59

**Number of figures:** 8 (main manuscript); 2 (supplementary manuscript)

^#^ These authors are equally contributed to this paper

* Correspondence to: Prof. Wei Zhang

Department of Pharmacology,

Pharmacy College, Nantong University,

19 QiXiu Road, Nantong, Jiangsu, China 226001

E-mail: huanghezhi36020@126.com

Phone: 0086-0513-85051728

Fax: 0086-0513-85051858

**Supplemental Experimental Procedures**

**Tail suspension test**

The TST test was performed according to the methods described previously ([Jiang et al., 2012](#_ENREF_3)). Briefly, mice were suspended 50 cm above the floor for 6 min by adhesive tape placed approximately 1 cm from the tip of the tail 30 min after single injection. The duration of immobility was recorded during the last 4-min by an investigator blind to the study. Mice were considered immobile only when they hung passively and were completely motionless, and any mice that did climb their tails were removed from the experimental analysis.

**Open field test**

The open field test was performed according to the methods described previously ([Jiang et al., 2012](#_ENREF_3)). The mice were placed individually in the dark in a wooden box (100 × 100 × 40 cm) with the ﬂoor divided into 25 (5 × 5) squares. The apparatus was illuminated with a red bulb (50 W) on the ceiling. Mice were placed in the central sector 30 min after single injection, and the total number of squares entered was recorded for 5 min under dim light conditions by an investigator blind to the study. The open field arena was thoroughly cleaned after each trial.

**Intracerebroventricular infusions of K252a and anti-BDNF-antibody**

In this study, we blocked the BDNF-TrkB system using K252a and chicken anti-BDNF antibody, which has been shown to be neutralizing and specific for BDNF ([Chen et al., 2005](#_ENREF_1); [Zhu et al., 2010](#_ENREF_5)). Briefly, C57BL/6J mice were anaesthetized with pentobarbital sodium, and placed in a stereotaxic frame. The cannulas were implanted into the left lateral brain ventricle (– 0.2 mm anterior and 1.0 mm lateral relative to Bregma and 2.3 mm below the surface of the skull) ([Kleinridders et al., 2009](#_ENREF_4)). The cannula was cemented in place, and the incision was sutured. The animals were allowed to recover for 3 d before the experiments started. Osmotic minipumps designed to deliver 0.05 µl/min each day were filled with 50 µM K252a in ACSF/50% DMSO, ACSF/50% DMSO, 20 µg/ml chicken anti-BDNF neutralizing antibody, or 20 µg/ml chicken IgY in ACSF (ﬁnal volume, 3 µl/mouse). Each osmotic minipump was attached to a brain infusion cannula.

**Chronic social defeat stress**

Social defeat and avoidance testing were performed according to our previous report ([Jiang et al., 2013](#_ENREF_2)). C57BL/6 mice were exposed to a different CD1 aggressor mouse each day for 10 min over a total of 10 d. After the 10 min of contact, C57BL/6 mice were separated from the aggressor: the test mice were placed in an adjacent compartment of the same cage, separated by a plastic divider with holes, where they were exposed to chronic stress in the form of threat for the next 24 h. Non-defeated control mice were handled daily and housed opposite another C57BL/6. 24 h after the last session, all the defeated mice were housed individually. After that, all the animals (control mice and defeated mice) were received daily injections of vehicle/tested compounds for 14 d, or intracerebroventricularly handled first and then treated with vehicle/tested compounds for 14 d.

The day after the last injection, a two-trial social interaction test was used to assay avoidance behaviors ([Jiang et al., 2013](#_ENREF_2)). In the first 5-min trial (“target absent”), the test C57BL/6 mouse was allowed to explore freely a square-shaped open-field arena possessing a wire-mesh cage apposed to one side, with their movement tracked. During the second 5-min trial (“target present”), the mouse was reintroduced into this arena now containing an unfamiliar CD1 mouse within the cage. The duration in the interaction zone were obtained using Ethovision XT (Noldus, USA) software (in seconds). After each trial, the apparatus was cleaned with a solution of 70% ethanol in water to remove olfactory cues.

Then the sucrose preference test was performed, mice were given the choice to drink from two bottles in individual cages, one with 1% sucrose solution and the other with water ([Jiang et al., 2013](#_ENREF_2)). All animals were acclimatized for 2 consecutive days to two-bottle choice conditions before 2 additional days of choice testing. The position of the bottles was changed every 6 h to prevent possible effects of side preference in drinking behavior. Before the test, animals were deprived of food and water for 24 h, and were then exposed to pre-weighed bottles for 1 h with their position interchanged. Sucrose preference was calculated as a percentage of the consumed sucrose solution relative to the total amount of liquid intake.

**Western blotting analysis**

The experiment was conducted as we have described ([Jiang et al., 2012](#_ENREF_3)). The test mice were sacrificed 24 h after the last drug exposure. Bilateral hippocampi were rapidly dissected and homogenized in lyses buffer for 30 min. The homogenate was centrifuged at 12000 × g for 15 min, and supernatants were then collected. Protein concentration was estimated by Coomassie blue protein-binding assay (Jiancheng Institute of Biological Engineering, Nanjing, China). After denaturation, 30 μg of protein samples were separated by 10% SDS/PAGE gel and then transferred to nitrocellulose membranes (Bio-Rad, Hercules, CA, USA). After blocking with 5% nonfat dried milk powder/Tris-buffered saline Tween-20 (TBST) for 1 h, membranes were incubated overnight at 4°C with primary antibodies to extracellular signal-Regulated Kinase 1/2 (ERK1/2; 1:1000), phospho-ERK1/2 (pERK1/2; 1:1000; Santa Cruz, CA, USA); AKT (1:1000), phospho-AKT (pAKT; 1:1000; Cell Signaling, MA, USA); cAMP response element-binding protein (CREB; 1:500), phospho-CREB-ser133 (pCREB; 1:500; Cell Signaling, MA, USA); brain-derived neurotrophic factor (BDNF; 1:500; Epitomics, CA, USA), GAPDH (1:1000; Santa Cruz , CA, USA). The antigen-antibody complexes were visualized with goat anti-rabbit or goat anti-mice horseradish peroxidase-conjugated secondary antibodies (1:2000; Santa Cruz, CA, USA) by using enhanced chemiluminescence (ECL; Pierce, Rockford, IL, USA). The optical density of the bands was determined using Optiquant software (Packard Instruments BV, Groningen, Netherlands).

**Immunohistochemical Studies**

For hippocampal doublecortin (DCX) staining, the test mice were deeply anaesthetized with pentobarbital sodium and perfused transcardially with 4% paraformaldehyde in 0.01 M phposphate buffer 24 h after the last drug exposure. The brains were removed and postfixed for 24 h, then dehydrated with 30% sucrose solution. After that, coronal brain sections of hippocampus were cut at 25 µm with a freezing microtome (CM1900, Leica Microsystems, Wetzlar, Germany) and collected serially. The sections were sequentially treated with 0.3% Triton X-100 in 0.01 M PBS for 30 min and 3% BSA in 0.01 M PBS for 30 min. They were then incubated with diluted goat anti-DCX antibody (1:100; Cell Signaling, MA, USA) overnight at 4°C. The sections were subsequently exposed to fluorescenin isothiocyanate (FITC)-labeled horse anti-rabbit IgG (1:50; Pierce, Rockford, IL, USA) for 1 h. They were then washed in 0.01 M PBS and mounted on slides following dehydration, and coverslipped. Sections were visualized with confocal laser scanning system (FV500; Olympus, Tokyo, Japan). Examination of DCX-positive (DCX+) cells was conﬁned to the DG, especially in the granule cell layer (GCL), including the subgranular zone (SGZ) of the hippocampus that deﬁned as a two-cell body-wide zone along the border between the GCL and the hilus. Quantiﬁcations of DCX+ cells were respectively conducted from 1-in-12 series of hippocampal sections spaced at 300 μm and spanning the rostrocaudal extent of the DG bilaterally. Every DCX+ cell within the GCL and SGZ was counted.

For the NeuN+/Brdu+ double labeling, the test mice were injected with Brdu (4×75 mg/kg at 2-h intervals) during the last 2 d of the 14-d drug treatment. Mice were sacriﬁced after 4 weeks and brain sections were then produced. DNA denaturation was conducted by incubation for 2 h with 50% formamide/2×SSC at 65 °C, followed by 30 min incubation in 2 N HCl at 37 °C, and rinsing in 0.1 M boric acid buffer (pH 8.5) at room temperature. After DNA denaturation, sections were treated with 0.3% Triton X-100 in 0.01 M PBS for 30 min and 3% BSA in 0.01 M PBS for 1 h, and then incubated with mouse monoclonal anti-BrdU (2 µg/ml, Roche) and rabbit monoclonal anti-NeuN (1:500; Abcam, Cambridge, UK) overnight at 4°C. After washing, FITC-conjugated horse anti-rabbit IgG and rhodamine-conjugated goat anti-mouse IgG (1:50; Pierce, Rockford, IL, USA) were applied for 1 h at room temperature. Sections were then washed in 0.01 M PBS and mounted on slides following dehydration, and coverslipped. Examination of NeuN+/Brdu+ co-labeling cells was conﬁned to the DG. Quantiﬁcations of NeuN+/Brdu+ cells were respectively conducted from 1-in-12 series of hippocampal sections spaced at 300 μm and spanning the rostrocaudal extent of the DG bilaterally. Every NeuN+/Brdu+ cell within the GCL and SGZ was counted.

**Supplemental References**

Chen J, Zhang C, Jiang H, Li Y, Zhang L, Robin A[Katakowski M](http://www.ncbi.nlm.nih.gov/pubmed?term=Katakowski%20M%5BAuthor%5D&cauthor=true&cauthor_uid=15678129), [Lu M](http://www.ncbi.nlm.nih.gov/pubmed?term=Lu%20M%5BAuthor%5D&cauthor=true&cauthor_uid=15678129), [Chopp M](http://www.ncbi.nlm.nih.gov/pubmed?term=Chopp%20M%5BAuthor%5D&cauthor=true&cauthor_uid=15678129) (2005) Atorvastatin induction of VEGF and BDNF promotes brain plasticity after stroke in mice. J Cereb Blood Flow Metab 25:281-290.

Jiang B, Wang W, Wang F, Hu ZL, Xiao JL, Yang S, [Zhang J](http://www.ncbi.nlm.nih.gov/pubmed?term=Zhang%20J%5BAuthor%5D&cauthor=true&cauthor_uid=23260228), [Peng XZ](http://www.ncbi.nlm.nih.gov/pubmed?term=Peng%20XZ%5BAuthor%5D&cauthor=true&cauthor_uid=23260228), [Wang JH](http://www.ncbi.nlm.nih.gov/pubmed?term=Wang%20JH%5BAuthor%5D&cauthor=true&cauthor_uid=23260228), [Chen JG](http://www.ncbi.nlm.nih.gov/pubmed?term=Chen%20JG%5BAuthor%5D&cauthor=true&cauthor_uid=23260228) (2013) The stability of NR2B in the nucleus accumbens controls behavioral and synaptic adaptations to chronic stress. Biol Psychiatry 74:145-155.

Jiang B, Xiong Z, Yang J, Wang W, Wang Y, Hu ZL, [Wang F](http://www.ncbi.nlm.nih.gov/pubmed?term=Wang%20F%5BAuthor%5D&cauthor=true&cauthor_uid=22335772), [Chen JG](http://www.ncbi.nlm.nih.gov/pubmed?term=Chen%20JG%5BAuthor%5D&cauthor=true&cauthor_uid=22335772) (2012) Antidepressant-like effects of ginsenoside Rg1 are due to activation of the BDNF signalling pathway and neurogenesis in the hippocampus. Br J Pharmacol166: 1872-1887.

Kleinridders A, Schenten D, Konner AC, Belgardt BF, Mauer J, Okamura T, [Wunderlich FT](http://www.ncbi.nlm.nih.gov/pubmed?term=Wunderlich%20FT%5BAuthor%5D&cauthor=true&cauthor_uid=19808018), [Medzhitov R](http://www.ncbi.nlm.nih.gov/pubmed?term=Medzhitov%20R%5BAuthor%5D&cauthor=true&cauthor_uid=19808018), [Brüning JC](http://www.ncbi.nlm.nih.gov/pubmed?term=Br%C3%BCning%20JC%5BAuthor%5D&cauthor=true&cauthor_uid=19808018) (2009) MyD88 signaling in the CNS is required for development of fatty acid-induced leptin resistance and diet-induced obesity. Cell Metab 10:249-259.

Zhu XH, Yan HC, Zhang J, Qu HD, Qiu XS, Chen L, [Li SJ](http://www.ncbi.nlm.nih.gov/pubmed?term=Li%20SJ%5BAuthor%5D&cauthor=true&cauthor_uid=20861371), [Cao X](http://www.ncbi.nlm.nih.gov/pubmed?term=Cao%20X%5BAuthor%5D&cauthor=true&cauthor_uid=20861371), [Bean JC](http://www.ncbi.nlm.nih.gov/pubmed?term=Bean%20JC%5BAuthor%5D&cauthor=true&cauthor_uid=20861371), [Chen LH](http://www.ncbi.nlm.nih.gov/pubmed?term=Chen%20LH%5BAuthor%5D&cauthor=true&cauthor_uid=20861371), [Qin XH](http://www.ncbi.nlm.nih.gov/pubmed?term=Qin%20XH%5BAuthor%5D&cauthor=true&cauthor_uid=20861371), [Liu JH](http://www.ncbi.nlm.nih.gov/pubmed?term=Liu%20JH%5BAuthor%5D&cauthor=true&cauthor_uid=20861371), [Bai XC](http://www.ncbi.nlm.nih.gov/pubmed?term=Bai%20XC%5BAuthor%5D&cauthor=true&cauthor_uid=20861371), [Mei L](http://www.ncbi.nlm.nih.gov/pubmed?term=Mei%20L%5BAuthor%5D&cauthor=true&cauthor_uid=20861371), [Gao TM](http://www.ncbi.nlm.nih.gov/pubmed?term=Gao%20TM%5BAuthor%5D&cauthor=true&cauthor_uid=20861371) (2010) Intermittent hypoxia promotes hippocampal neurogenesis and produces antidepressant-like effects in adult rats. J Neurosci 30:12653-12663.

**Supplemental Figure Legends**

**Supplementary Figure S1.** Acute TMP treatement has no significant antidepressant effects in the CSDS model of depression. C57BL/6J mice were exposed to defeat stress for 10 d, and received one injection of vehicle, or TMP (10, 20 mg/kg). Behavioral tests were conducted 24 h after the injection. (A) The social interaction in CSDS + TMP mice was similar to that in CSDS + vehicle mice. (B) The sucrose consumption in CSDS + TMP mice was also similar to that in CSDS + vehicle mice. Data are expressed as means ± S.E.M. (n = 10); *******P* < .01; n.s., no significance. Comparison was made by two-way ANOVA followed by post-hoc Bonferroni’s test.

**Supplementary Figure S2.** The antidepressant actions of TMP occur independent of serotonergic system. (A) Depleting serotonin with the tyrosine hydroxylase inhibitor PCPA before TMP administration did not eliminate the antidepressant effects of TMP in the FST. (B) PCPA pretreatment had no influence on the antidepressant effects of TMP in the TST. (C) CSDS-treated mice were co-injected with TMP and PCPA for 14 d, behavioral tests were then performed. In the sucrose preference test, TMP treatment continued to produce antidepressant effects following serotonin depletion. (D) In the social interaction test, mice treated with both TMP and PCPA did not differ significantly from TMP-treated mice. Results are expressed as means ± S.E.M. (n = 10); *******P* < .01; n.s., no significance. Comparison was made by one-way ANOVA followed by post-hoc LSD test.
